# Effect of using a trephine to extract bone at the insertion point of the intramedullary nails and graft bone at the fractured ends in the treatment of tibial shaft fractures with intramedullary nail

**DOI:** 10.12669/pjms.41.4.10259

**Published:** 2025-04

**Authors:** Zhaoning Xu, Guoping Cai, Xianwei He, Defang Li

**Affiliations:** 1 Zhaoning Xu, MM, Orthopedics Department, Fudan University Jinshan Hospital, 1508 Longhang Road, Jinshan District, Shanghai 201508, China; 2 Guoping Cai, MD, Orthopedics Department, Fudan University Jinshan Hospital, 1508 Longhang Road, Jinshan District, Shanghai 201508, China; 3 Xianwei He, MM, Orthopedics Department, Fudan University Jinshan Hospital, 1508 Longhang Road, Jinshan District, Shanghai 201508, China; 4 Defang Li, MD, Orthopedics Department, Fudan University Jinshan Hospital, 1508 Longhang Road, Jinshan District, Shanghai 201508, China

**Keywords:** Bone grafting, Intramedullary nail, Tibial shaft fracture

## Abstract

**Objective:**

To explore the effect of using a trephine to extract bone at the insertion point of the intramedullary nails (IMN) and graft bone at the fractured ends in the treatment of tibial shaft fractures with IMN.

**Methods:**

In this single-center retrospective study, patients who underwent treatment for tibial shaft fracture using IMN between January 2016 and December 2019 at Fudan University Jinshan Hospital were retrospectively analyzed. There were two groups. In the study group, a trephine was used to extract bone chips at the insertion point of the IMN, which were grafted at the fractured ends. The control group comprised sex- and age-matched patients with similar fracture location and classification, but without bone graft at the fractured ends. The surgery time, fracture healing time, and frequency of IMN dynamic surgery were compared between the two groups. Furthermore, the Johner-Wruhs score, WHO-QOL score, and knee joint range of motion at six and 12 months after surgery were compared.

**Results:**

Both groups had 99 matched patients (69 male and 30 female). The median healing time was significantly shorter in the study group (*z*=-2.86, *p*=0.004). Five cases (5.05%) in the study group and 10 cases (10.10%) in the control group underwent IMN dynamic surgery; the between-group difference in this respect was not statistically significant (c^2^=1.803, *p*=0.179). There was no significant between-group difference regarding the other parameters.

**Conclusions:**

The surgical technique can promote healing of tibial shaft fractures with no adverse effect on postoperative functional recovery.

## INTRODUCTION

Tibial shaft fracture is the most common tubular fracture^1^, and is typically caused by injury sustained during road traffic accidents and sporting activities.^2^ Fractures at the middle and lower 1/3 of the tibial shaft often damage the nutrient artery. Furthermore, most of the middle and lower tibia have no muscle attachment, and a long segment is directly located under the skin, increasing the risk of delayed union or nonunion of fractures.^3^

Intramedullary nail (IMN) fixation is the gold standard for the treatment of tibial shaft fracture.^4^ Compared to a plate, the IMN offers the advantages of strong torsion resistance, low infection rate, and good callus growth.^5^ Sometimes a satisfactory closed reduction is not achievable in some cases, and it can be difficult to accurately insert the guide pin. Such situations necessitate open reduction. However, this further impairs the blood supply of the fracture ends. The reported incidence of nonunion after IMN fixation for tibial shaft fractures was around 12.7%.^6^

**Fig.1:**
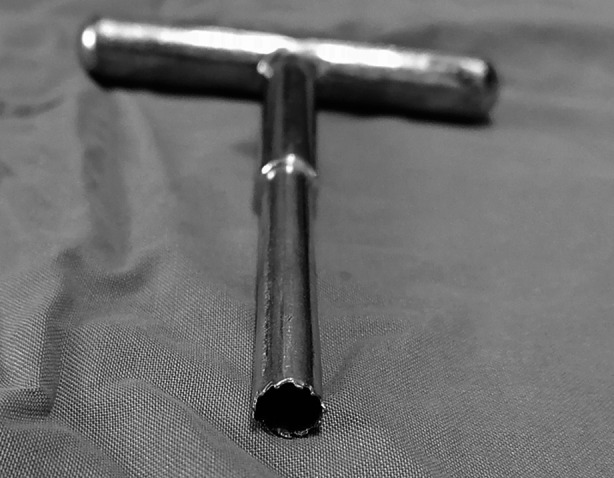
A trephine

The occurrence of fracture nonunion or delayed union necessitates additional surgery to dynamize the IMN and obtain the autologous iliac bone graft. In some cases, it may be necessary to replace the original IMN. This greatly increases the pain and financial burden on patients, and prolongs the recovery time. In addition, autologous iliac bone graft causes new trauma to the patient. During the surgery of tibial shaft fractures with IMN, some surgeons used a trephine (Fig.1), instead of an opener, to open the entry point of the tibial IMN for bone extraction and bone grafting at the fracture ends.

However, there is no robust evidence of the efficacy of this surgical method in promoting fracture healing after tibial shaft fracture surgery. Therefore, the purpose of this study was to explore whether bone extraction at the insertion point of the tibial IMN and bone grafting at the fracture ends can promote fracture healing and reduce the rate of secondary surgery in the treatment of tibial shaft fractures with IMN.

## METHODS

This was a single-center retrospective study. Patients were identified from the medical records using the ICD-10 codes. Patients who underwent treatment for tibial shaft fracture using IMN between January 2016 and December 2019 at Fudan University Jinshan Hospital were retrospectively analyzed.

### Ethical Approval:

The study was approved by the Ethics Committee of Fudan University Jinshan Hospital (JIEC 2022-S07). The validity of the approval was from February 23, 2022 to 22nd February 2023.

### Inclusion criteria:

- The first diagnosis was tibial shaft fracture (ICD-10: S82.200), tibiofibular shaft fracture (ICD-10: S82.201), tibial fracture (ICD-10: S82.202), or tibiofibular closed fracture (ICD-10: S82.203).
- The operation was open reduction and intramedullary nail fixation of tibial fracture (ICD10: 79.3600×015).
- The operation was conducted between January 2016 and December 2019.

### Exclusion criteria:

- Concomitant presence of fractures in other parts.
- Patients with concomitant craniocerebral trauma or thoracoabdominal organ injury.
- Open fractures.
- Cases with incomplete follow-up.

### Case grouping:

For patients in the study group, a trephine was used to extract bone chips at the insertion point of the IMN and place bone grafts at the fractured ends. Patients in the control group comprised of sex- and age-matched (a difference of within 10 years) patients with similar fracture location and classification, but without bone graft at the fractured ends.

### General information:

Based on the inclusion and exclusion criteria, 121 patients were selected in the study group. Of these, 99 patients (69 males and 30 females) were successfully matched to patients in the control group. According to the AO Foundation and Orthopedic Trauma Association classification, there were 21 cases of type A1, 15 cases of type A2, 12 cases of type A3, 22 cases of type B1, 13 cases of type B2, 15 cases of type B3, and one case of type C1. The average age of patients in the study group was 53.04 ± 13.50 years (range: 18-86), with the left limb involved in 53 cases and the right limb involved in 46 cases. The average age of patients in the control group was 49.79 ± 12.43 years (range: 22-80) with the left limb involved in 50 cases and the right limb involved in 49 cases.

### Surgical methods:

All patients were administered general anesthesia in the supine position. During the operation, the hemostasis of the affected limb was achieved using a pneumatic tourniquet. Open reduction was performed for tibial fractures. The skin and patella tendon were cut through a conventional longitudinal incision under the patella. For patients in the study group, the insertion point of the IMN was exposed in front of the tibial plateau. Then, a trephine was used to obtain a long strip of cancellous bone. After the insertion of the IMN, the cancellous bone strip was cut and implanted at the fracture end. Then, the incision was sutured layer by layer. Patients in the control group were only treated with open reduction and IMN fixation of tibial fractures, without bone removal and grafting.

### Postoperative treatment:

After awakening from general anesthesia, the patients were encouraged to actively engage in ankle and quadriceps isometric contraction without weight bearing. Antibiotics were administered within 24 hours after the operation to prevent infection. The stitches were removed two weeks after the surgery. At six weeks after the surgery, weight-bearing activities were gradually initiated with the affected limb. X-ray examinations were regularly performed after the surgery to observe the fracture healing. At six months after the surgery, cases with no signs of fracture healing underwent IMN dynamic surgery.

### Statistical analysis:

The surgical time, fracture healing time, and cases of IMN dynamic surgery were compared between the two groups. In addition, the Johner-Wruhs score, WHO-QOL score, and knee joint range of motion (ROM) at six and 12 months after the surgery were compared. Statistical analysis was performed using the SPSS v22.0 software (IBM). Between-group differences regarding continuous variables were assessed using Mann-Whitney *U*-test when variables were skew distribution and paired Student’s *t*-test when variables were normally distributed. Chi-square test was performed on categorical variables. *P* values < 0.05 were considered indicative of statistical significance.

## RESULTS

There were no significant differences between the two groups with respect to age, affected side (left or right), or body mass index (Table-I). Furthermore, there was no significant between-group difference with respect to postoperative Johner-Wruhs scores at six months (χ*2*=2.945, *p*=0.086) and 12 months (χ*2*=0, *p*=1) after the surgery (Table-II).

**Table-I:** General information of patients in the study and control groups.

Group	Sample size	Age	Side		BMI
			Left	Right	
Study group	99	53.04 ± 13.50	53	46	24.63 ± 2.86
Control group	99	49.79 ± 12.43	50	49	24.15 ± 2.33
Statistic	-	*t* = 1.760	c^2^ = 0.782		*t* = 1.280
*p*-value	-	0.08	0.67		0.20

*Notes:* BMI, body mass index.

**Table-II:** Comparison of postoperative Johner-Wruhs scores between the two groups (n, %).

Group	Johner-Wruhs score			
	6 months postoperative		12 months postoperative	
	Excellent	Good	Excellent	Good
Study Group	87 (87.88)	12 (12.12)	90 (90.90)	9 (9.10)
Control Group	78 (78.79)	21 (21.21)	90 (90.90)	9 (9.10)
Statistic	c^2^ = 2.945		c^2^ = 0	
*p*-value	0.086		1.000	

Moreover, the median surgery time was not significantly different between the two groups (*z*=-1.626, *p*=0.104; Table-III). However, the median healing time in the study group (five months [range: 5–6]) was significantly shorter than that in the control group (six months [range, 5-7]) (*z*=-2.86, *p*=0.004). Five cases (5.05%) in the study group and 10 cases (10.10%) in the control group underwent IMN dynamic surgery. The rate of IMN dynamic surgery was not significantly different between the two groups (χ*2*=1.803, *p*=0.179; Table-III). After the dynamic surgery, all fractures eventually healed.

**Table-III:** Comparison of surgical time, fracture healing time, and frequency of dynamic surgery in the two groups.

Group	Surgical time	Fracture healing time	Dynamic rate
Study group	106.00 (95.00–114.00)	5 (5–6)	5 (5.05)
Control group	102.00 (96.00–110.00)	6 (5–7)	10 (10.10)
Statistic	*z* = -1.626	*z* = -2.860	c^2^ = 1.803
*p*-value	0.104*	0.004*	0.179**

*Notes:*

* Rank-sum test;

** Chi-square test.

There was no significant between-group difference in terms of the WHO-QOL scores for any of the four domains at six and 12 months after surgery (Table-IV). Furthermore, there was no significant between-group difference in terms of knee joint ROM at six months (*t*=-1.528, *p*=0.13) and 12 months (*t*=-0.755, *p*=0.452) after surgery (Table-IV).

**Table-IV:** Comparison of postoperative WHO-QOL scores and knee joint ROM between the two groups.

Time	Item	Study group	Control group	Statistic	p-value
6 months postoperative	Physical	89.94 ± 5.96	90.87 ± 4.31	-1.392	0.167
	Psychological	93.56 ± 3.33	93.77 ± 3.22	-0.439	0.662
	Social relationship	86.62 ± 4.88	85.86 ± 4.68	1.100	0.274
	Environment	88.70 ± 2.63	88.04 ± 2.64	1.680	0.096
	Knee ROM	125.96 ± 4.00	127.03 ± 5.71	-1.528	0.13
12 months postoperative	Physical	92.32 ± 4.06	93.22 ± 3.33	-1.916	0.058
	Psychological	93.77 ± 3.17	94.11 ± 2.59	-0.816	0.417
	Social relationship	88.72 ± 4.01	89.23 ± 4.17	-0.865	0.389
	Environment	90.44 ± 2.56	90.94 ± 2.50	-1.388	0.168
	Knee ROM	129.72 ± 2.74	130.11 ± 4.45	-0.755	0.452

*Notes:* ROM, range of motion.

## DISCUSSION

This research highlights a new surgical method capable of facilitating the healing process of tibial shaft fractures, thus making some progress in the field of fracture treatment. It can promote patient recovery and reduce the economic burden on patients and society. This study used paired grouping in case selection to minimize the impact of confounding factors.

The results of our study showed that during the surgery of tibial shaft fracture with IMN, bone extraction at the insertion point of the IMN and bone grafting at the fracture end can promote fracture healing. Meanwhile, it will not affect the postoperative functional recovery of patients. Giannoudis et al. proposed the diamond concept, a framework for a successful bone repair response that accords equal importance to mechanical stability and the biological environment for fracture healing.^7^ It refers to the availability of osteogenic cells, osteoconductive scaffolds, the mechanical environment, and growth factors.^7^ The cancellous bone of the proximal tibia is rich in bone marrow tissue and contains a variety of osteogenic factors. Bone grafting can eliminate the gap between the fracture ends, eliminate bone defects, provide a good scaffold for fracture healing, and prevent the growth of soft tissue into the fracture ends, promoting fracture healing.

The operation method presented in this paper can achieve bone grafting at the time of internal fixation of fracture, which can create favorable conditions for fracture healing to the greatest extent. The reamer irrigator aspirator system requires a special set of instruments for bone removal, and there are some complications, such as bone cortex perforation and heavy bleeding.^8^ The bone extraction method described in the present study merely requires the use of a trephine. This is easy to operate, does not need special instruments, and does not prolong the surgery time. The bone tunnel that formed after bone extraction by the trephine was immediately filled with IMN, preventing additional bone defects.

In this study, there was no significant difference between the two groups in terms of postoperative rehabilitation and knee ROM, indicating that this method of bone extraction does not affect the postoperative recovery of lower limb function. However, there is a limitation to the application of this method. It is not applicable to the suprapatellar approach IMN internal fixation. And there were some studies which found that the suprapatellar approach can significantly reduce the degree of pain, promote the recovery of patients with knee joint involvement and will not lead to rotational malalignment.^9^,^10^

In our study, we found that the fracture healing time in the study group was significantly shorter than that in the control group, but there was no difference in the dynamic rate of intramedullary nailing between the two groups. This may because most cases in the present study were Type-A and Type-B. Further study is needed to determine whether this surgical method will reduce the dynamic rate of intramedullary nailing for type C tibial shaft fractures. There was a study found that type C tibial shaft fractures are at a high risk of delayed union after IMN treatment.^11^ In other words, for Type-A and Type-B fractures, this surgical method can shorten the healing time, but this may not affect the outcome of fracture healing. Therefore, the investigators do not recommend exposure of the fracture end only for bone grafting. If the fracture end has to be exposed due to poor reduction or other reasons, bone grafting can be performed at the same time.

### Limitations

This is a single center retrospective study. This could potentially introduce an unavoidable selection bias. Further prospective studies are needed. These studies may provide more comprehensive data on the effect of using a trephine to extract bone at the insertion point of the intramedullary nails and graft bone at the fractured ends in the treatment of tibial shaft fractures with intramedullary nail.

## CONCLUSION

In the treatment of tibial shaft fracture with IMN, bone extraction at the insertion point of the IMN and bone grafting at the fracture end can promote fracture healing without affecting the postoperative functional recovery of patients.

## References

[1] Kojima KE, Ferreira RV (2011). Tibial Shaft Fractures. Rev Bras Ortop.

[2] Madadi F, Vahid FM, Eajazi A, Daftari BL, Madadi F, Nasri LM (2010). Epidemiology of adult tibial shaft fractures:a 7-year study in a major referral orthopedic center in Iran. Med Sci Monit.

[3] McMillan TE, Johnstone AJ (2017). Technical considerations to avoid delayed and non-union. Injury.

[4] Zelle BA, Boni G (2015). Safe surgical technique:intramedullary nail fixation of tibial shaft fractures. Patient Saf Surg.

[5] Jindal K, Neradi D, Sodavarapu P, Kumar D, Shetty A, Goni V (2021). Intramedullary Nailing Versus Plating for Proximal Tibia Fractures:A Systematic Review and Meta-analysis. Indian J Orthop.

[6] Guo J, Zha J, Di J, Yin Y, Hou Z, Zhang Y (2021). Outcome Analysis of Intramedullary Nailing Augmented with Poller Screws for Treating Difficult Reduction Fractures of Femur and Tibia:a Retrospective Cohort Study. Biomed Res Int. 2021.

[7] Giannoudis PV, Einhorn TA, Marsh D (2007). Fracture healing:the diamond concept. Injury.

[8] Laubach M, Weimer LP, Blasius FM, Hildebrand F, Kobbe P, Hutmacher DW (2023). Complications associated using the reamer-irrigator?-aspirator (RIA) system:a systematic review and meta-analysis. Arch Orthop Trauma Surg.

[9] Zhu Z, Wang Z, Zhou P, Wang X, Guan J (2021). Comparison of clinical efficacy of suprapatellar and infrapatellar intramedullary nailing in treating tibial shaft fractures. Pak J Med Sci.

[10] Alderlieste DS, Cain ME, Van der Gaast N, Verbakel J, Edwards B, Jaarsma EH (2024). Prevalence of Rotational Malalignment After Infrapatellar Versus Suprapatellar Intramedullary Nailing of Tibial Shaft Fractures. JB JS Open Access.

[11] Kawasaki N, Takegami Y, Sakai R, Todoroki K, Uemi R, Imagama S (2022). Prediction of delayed union of tibial shaft fracture treated with intramedullary nailing:multicenter-study analysis and literature review -the TRON study. Eur J Orthop Surg Traumatol.

